# Multiscale Estimation of Binding Kinetics Using Brownian Dynamics, Molecular Dynamics and Milestoning

**DOI:** 10.1371/journal.pcbi.1004381

**Published:** 2015-10-27

**Authors:** Lane W. Votapka, Rommie E. Amaro

**Affiliations:** Department of Chemistry and Biochemistry and National Biomedical Computation Resource, University of California, San Diego, San Diego, California, United States of America; George Mason University, UNITED STATES

## Abstract

The kinetic rate constants of binding were estimated for four biochemically relevant molecular systems by a method that uses milestoning theory to combine Brownian dynamics simulations with more detailed molecular dynamics simulations. The rate constants found using this method agreed well with experimentally and theoretically obtained values. We predicted the association rate of a small charged molecule toward both a charged and an uncharged spherical receptor and verified the estimated value with Smoluchowski theory. We also calculated the k_on_ rate constant for superoxide dismutase with its natural substrate, O_2_^−^, in a validation of a previous experiment using similar methods but with a number of important improvements. We also calculated the k_on_ for a new system: the N-terminal domain of Troponin C with its natural substrate Ca^2+^. The k_on_ calculated for the latter two systems closely resemble experimentally obtained values. This novel multiscale approach is computationally cheaper and more parallelizable when compared to other methods of similar accuracy. We anticipate that this methodology will be useful for predicting kinetic rate constants and for understanding the process of binding between a small molecule and a protein receptor.

## Author Summary

We estimated the k_on_ rate constant of four biochemically relevant ligand-receptor systems using milestoning theory. All results closely resemble experimentally and theoretically determined results, indicating that this technique may be applied toward accurate estimation of binding rate constants for additional ligand-receptor systems of biomedical interest.

This is a *PLOS Computational Biology* Methods paper

## Introduction

Estimating kinetics is an important and challenging task in computational biophysics. The kinetic rate constants of ligand-receptor interactions, in particular the k_on_ and k_off_ values, play an important role in enzymology[1] and drug discovery[2]. Kinetic rate constants of ligand-receptor association and dissociation are important determinants of drug efficacy[2], and the optimization of these quantities is an important problem in medicinal chemistry. Although these values may often be measured experimentally, an accurate computational estimate would be attractive in cases where experimental measurement is expensive or difficult. In addition, advances in computational power, particularly in parallel computing, offer great potential for methods that take advantage of the vast and increasing power of computation.

As indicated in Eq 1, ligands typically bind to receptors according to a second order reaction process with a rate constant of k_on_. Unless a nonreversible reaction occurs, ligands typically unbind from their receptors according to a first order process with a rate constant of k_off_.

$$R+L\;\overset{k_{on}}{\underset{k_{off}}{⇄}}\;RL$$ (1)

A number of computational techniques exist to predict rate constants. The timescale of kinetic events vary wildly in biomolecular systems, and can extend between 10^8^ events per second to less than 1 event per hour[1] for a single reaction event at physiological concentrations of reactants. For computational methods that estimate kinetic quantities, there is typically a high correlation between accuracy and computational cost. Explicit all-atom molecular dynamics (MD) is one approach to estimate the k_on_ between a protein and a small molecule[3–6]. Though it offers a relatively high degree of accuracy, this technique involves extensive cyberinfrastructure overhead or access to specialized hardware such as the Anton machine[7]. To our knowledge, the longest MD simulations to date are limited to the low millisecond range[8].

Various theories and algorithms offer cheaper alternatives to making kinetic approximations using brute-force, all-atom explicit MD simulations. Examples include two closely related techniques: Markov state models (MSM)[9–17] and milestoning[18–23] among many others.

Brownian dynamics (BD) is a simulation method used to model macromolecular diffusion in an aqueous solvent[24]. Compared to MD simulations of intermolecular encounters, BD simulations typically require far less computation to simulate an association event. Due to various approximations, including rigid body dynamics, reduced point-charge interactions, implicit solvent, and a relatively large timestep, millions of protein/small molecule binding or association events can be simulated in 24 hours using modest parallelization. However, the approximations and assumptions made when using BD to simulate molecular binding can also introduce inaccuracies. BD can be used alone to model ligand association[25]. However, an accurate recovery of experimentally determined observables related to a binding process frequently requires additional models to approximate physical effects due to solvation shells and polarization, solvent entropic effects, and solute internal degrees of freedom. Some schemes to include these factors in BD simulations have been implemented[26–29].

Methods for combining the speed of rigid body BD simulations with the precision of all-atom MD simulations to predict kinetics have been used in the past. In a technique invented by Luty, El Amrani, & McCammon, the k_on_ of superoxide dismutase (SOD) with its natural substrate O_2_^−^ was estimated by partitioning space into a region close to the binding site for simulation with MD, and a region far from the binding site where simulation with BD was more appropriate[30,31]. The statistics of each were combined into a k_on_ estimate using a MSM.

Although Luty et. al.’s original method dramatically decreased the cost to estimate binding kinetics compared to brute-force MD, a number of optimizations can be made to the procedure. Though proportionally smaller, the MD regime was disproportionally more expensive than the BD in Luty et. al.’s initial implementation. In this work, we used milestoning theory instead of a MSM to utilize the transition probabilities and incubation times between states. We modified Luty et. al.’s method by further partitioning the MD component with additional milestones. We also used a first hitting point distribution (FHPD) as the starting phase space points for the milestoning trajectories rather than an equilibrium distribution[20,22], a required procedure in milestoning theory. It is interesting to note that Luty et. al.’s method was remarkably similar to milestoning. Their use of surface states in phase space and a transition matrix to represent traversal between the states was somewhat prescient. However, Luty et. al. did not go so far as to integrate time information into the method to estimate mean first passage times (MFPT), nor did they did use FHPDs. Milestoning proper came later[18] and the formalism has since been extensively developed by others[18–22]. A milestoning model is very similar to a MSM; so much so that milestoning techniques have been used to perform MSM calculations[32], and a number of papers provide extensive comparisons of the two approaches[19,33,34].

**Fig 1:**
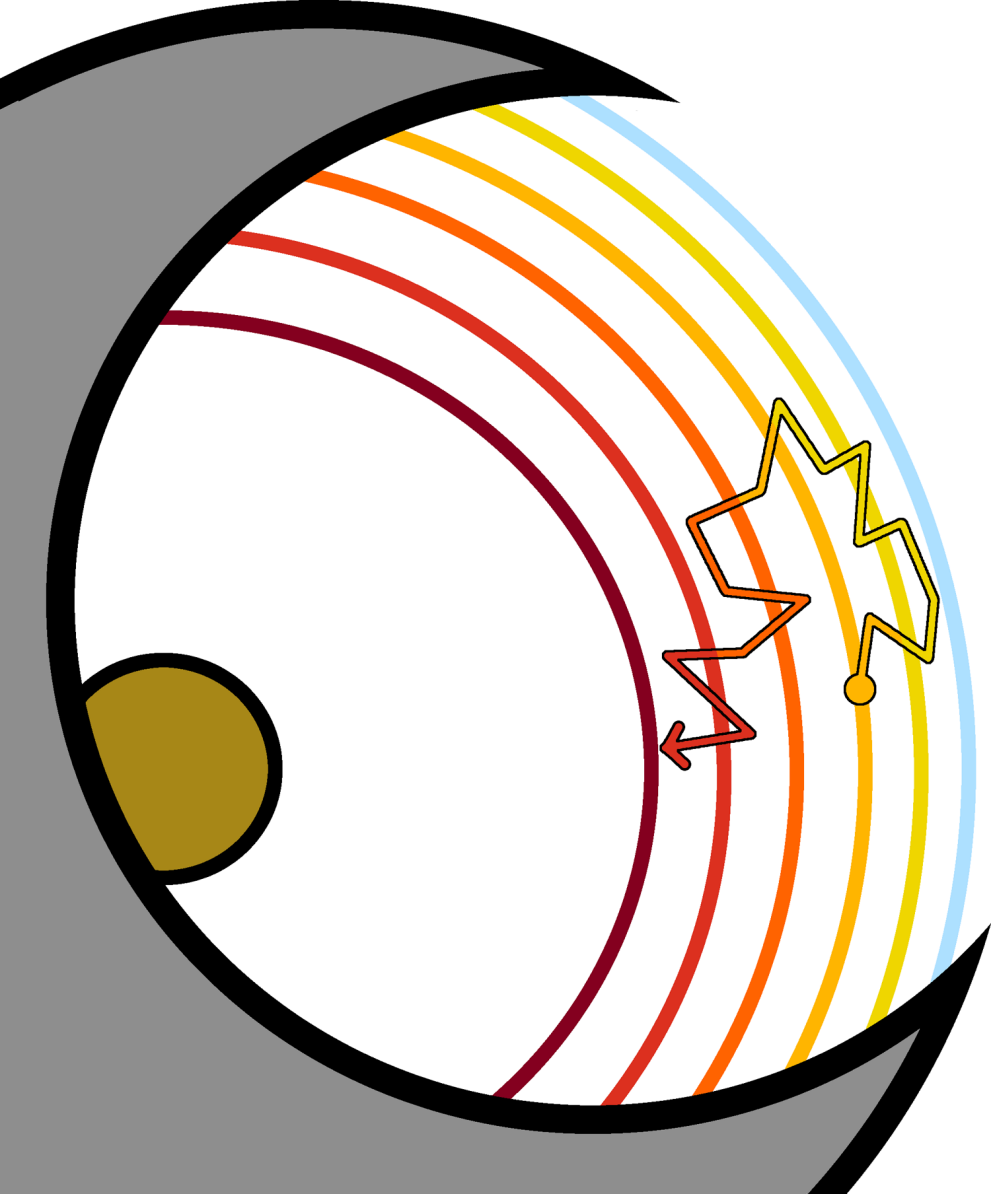
A cartoon depiction of a hypothetical path taken by a ligand as it diffuses in the vicinity of its binding site in the MD simulation regime. As the ligand travels, it crosses a series of milestones. Upon crossing, the ligand is considered to be in the crossed milestone’s state until it diffuses across a different milestone. The trajectory is terminated when the ligand crosses the “binding surface”, where it is considered bound, or when it crosses the BD surface, thus exiting the MD simulation regime.

In addition to repeating the analysis of SOD made by Luty et. al. with our new method, we also estimated k_on_ values for three additional systems. We calculated k_on_s for two simple, theoretically verifiable “spherical receptor” systems: the rate that a Na^+^ particle crosses an uncharged sphere of radius 6.0Å, and the rate the same particle crosses a charged sphere of radius 6.0Å (Fig 1). We also estimated the k_on_ of binding between the N-domain of Troponin C (TnC) and its natural substrate Ca^2+^. Since experimentally measured k_on_s existed for each of the two protein systems mentioned above, we attempted to closely recreate the experimental conditions within our simulations and subsequently recapture the correct k_on_s to validate our methods. Armed with this technique, one can make new attempts to estimate kinetic values for biologically or pathogenically interesting systems.

### Theory

**Molecular dynamics** is a simulation technique that uses Newton’s or Langevin’s equations of motion in combination with a specified molecular bond structure, parametrized force fields, and a starting conformation of atomic positions and velocities in order to propagate the dynamics of atoms within a molecular system. Ensembles of conformations or trajectories can be sampled to estimate thermodynamic or kinetic quantities[20,35,36].

#### Brownian dynamics

In addition to MD, BD simulation is another technique that can be used to model macromolecular diffusion in an aqueous solvent[24,25,37,38]. BD can also be used to model the association of biomolecules in solution[39]. BD simulations rely on the assumptions inherent to the theory of Brownian motion[24,39,40]. In the simplest form of BD, these assumptions include: a solvent whose atoms may be approximated by a dielectric and ionic continuum and whose hydrodynamic properties can be described using diffusion coefficients or tensors, solute molecules that can be adequately represented as rigid bodies, and forces that can be reduced to electrostatics, steric hindrances, and other inter-solute interactions. BD simulations are propagated according to the general equation of Brownian motion[27] (Eq 2) which has been derived from the N-particle Fokker-Planck Equation[41,42].

$$d\left(\begin{array}{c} x_{i}\\ \varphi_{i} \end{array}\right)=\frac{dt}{k_{B}T}D\cdot \left(\begin{array}{c} F_{i}\\ {Τ}_{i} \end{array}\right)+\sqrt{2dt}S\cdot w+\nabla \cdot Ddt$$ (2)

Where *i* is the index of a particle in the system. The values ***x***_***i***_, *φ*_*i*_, ***F***_***i***_, ***T***_***i***_ are the position, rotation, force, and torque of particle *i* respectively, **D** is the diffusion tensor, **S** is matrix square root of **D**, **w** is a random vector whose components are Gaussian variables with unit variance and zero mean. The Northrup-Allison-McCammon algorithm[25] solves these equations numerically, and can be used to propagate timesteps within a BD simulation.

When BD is used to estimate the k_on_ of a ligand-receptor association reaction, the k_on_ rate constant can be split into two terms. (Eq 3)

$$k_{on}=k_{b}\beta$$ (3)

Where *k*_*b*_ is the rate of diffusion of the ligand to a spherical surface of radius *b* (b-surface) centered on the ligand. The *k*_*b*_ can be calculated by using Eq 4.

$$k_{b}=4\pi {\left[\overset{\infty }{\underset{\text{b}}{\int }}\frac{\text{exp}\left(\text{U}\left(\text{r}\right)/k_{B}T\right)}{r^{2}D\left(r\right)}dr\right]}^{-1}$$ (4)

Where *U(r)* is the effective potential energy between the receptor and the substrate at a distance *r* from the center of the receptor, *k*_*B*_ is Boltzmann’s constant, and *T* is temperature, and *D(r)* is the spatially-varying diffusion coefficient. *β* is the probability that a ligand located on the b-surface will continue on to react with the enzyme rather than escaping to an infinite distance. Normally, *β* can be determined by running BD simulations started from random locations on the b-surface and then counting the proportion of trajectories that lead to binding. In this study, *β* was determined by combining BD with MD using milestoning.

#### Milestoning theory

Milestoning computationally models the kinetics as well as the thermodynamics of chemical processes, with the benefit of extensive parallelizability [18,19,22,43]. Using milestoning techniques, the stationary flux distribution **q** and the probability distribution **p** can be found across a reaction coordinate along which a number of milestones have been defined. Milestoning can also be used to find the mean first passage time (MFPT) of a transport process starting from one milestone and ending at another. The methods within milestoning theory provide a flexible approach to investigate a wide range of dynamics, including non-equilibrium conditions [21,23] and has been applied in a variety of contexts [23,32,33,44,45]. Milestoning does not rely on any assumption concerning system damping [18], and thus can be applied to Newtonian, Langevin, and Brownian systems alike [18,19,22].

In our implementation, we defined a number of concentric spherical surfaces in phase space that encircle the binding site on each receptor. These surfaces in phase space are termed “milestones” and are roughly perpendicular to the reaction coordinate (Fig 1).

In a typical milestoning procedure, unbiased simulations are initiated from a set of equilibrium distributions along the milestones, which one obtains using umbrella sampling. Each of these simulations is independent from the others, and the ligand center of mass is positioned at or very near the milestoning surface. As the simulations progress, transitions between milestones are recorded to construct a proper FHPD across the milestones, and then used to construct a transition kernel matrix with elements **K**, whose entries describe the probability that a ligand in one of the milestones will subsequently transition to another. An incubation time vector 〈*t*〉 is also obtained by determining the average time the system takes to transition from each milestone to an adjacent one. Given these quantities, stationary fluxes **q** across each milestone, the probability distribution **p**, and MFPT 〈*τ*〉 are found using eqs 5–7.

$$q_{stat}\left(I-K\right)=0$$ (5)

$$p_{i,\;stat}=q_{i,stat}\cdot t_{i}$$ (6)

$$\langle \tau \rangle =p\cdot {\left(I-\tilde{K}\right)}^{-1}\langle \text{t}\rangle$$ (7)

where **I** is the identity matrix, *i* is the index of a particular milestone, and $\tilde{K}$ is the transition kernel with one or more absorbing ‘sink’ states.

Milestoning theory, as well as the method employed by Luty et. al., defines states using surfaces in phase space. The current state of the simulated system is the surface that has been most recently crossed. Each of the surfaces must be sufficiently far apart from one another in order to ensure that velocity is decorrelated between transitions. In our implementation, we defined our milestones as concentric spheres in order to closely approximate isosurfaces of the committor function. For a rigorous discussion of ‘surface’ states and their requirements and assumptions, the reader is referred to additional publications on milestoning theory[18–20,22].

In our implementation, all trajectories used to populate the statistics in the milestoning model are started from FHPDs calculated on each of the surface states. The FHPD represents the distribution of system conformations that have just crossed a surface state and that had previously been in a different state. The difference between the FHPD and an equilibrium distribution is that the latter also includes conformations whose last crossing event was the same as the current state. A trajectory is started from the FHPD and allowed to propagate according to the simulation dynamics, crossing surfaces as it diffuses (Fig 1). If the trajectory ever crosses the surface of a sink state, such as the bound state or a state leading to another simulation regime, the trajectory is halted. As surfaces are crossed, the counts are tallied to construct the transition matrix **K** and the average incubation time vector 〈*t*〉. In our implementation, *β* was found by summing the stationary fluxes of all bound milestones according to Eq 8.

$$\beta =\underset{z}{\Sigma }q_{z,stat}$$ (8)

Where *z* is the index of a milestone that represents a bound state.

Error estimation of computed values were made using a Monte Carlo method to sample matrix distributions defined in milestoning[22,34] similar to one used in MSM theory[46]. Details of error estimation are outlined in the Supplementary Information (S1 Text)(S1 Fig).

#### Theoretical determination of k_on_

The flux rate *k(r)* of a particle across a sphere of radius *r* may be estimated analytically for some simple systems. The value *k(r)* is equivalent to k_on_ if a sphere of radius *r* is modeled as a binding surface. In the uncharged spherical receptor system, there are no average forces on the substrate and the *k(r)* can be obtained by solving the Smoluchowski equation[47].

k(r)=4πrD (9)

Where *r* is the radius of the reacting sphere, and *D* is the diffusion coefficient of the substrate. The *k(r)* can also be calculated for systems with centrosymmetric forces by solving the Smoluchowski equation in spherical coordinates[47,48]. The result is expressed as Eq 4. Assuming a constant diffusion coefficient and that the effective potential energy is defined by Coulomb’s Law in a uniform dielectric, Eq 4 can be reduced and solved exactly for the charged spherical receptor system (Eq 10):

$$k\left(r\right)=-\frac{DQ_{c}Q_{s}}{\left[1-exp\left\{\frac{Q_{c}Q_{s}}{4\pi \varepsilon_{0}\varepsilon_{r}k_{B}Tr}\right\}\right]\varepsilon_{0}\varepsilon_{r}k_{B}T}$$ (10)

Where *Q*_*s*_ is the charge of the diffusing particle, *Q*_*c*_ is the charge in the center of the receptor sphere, *ε*_*0*_ is the permittivity of a vacuum, and *ε*_*r*_ is the dielectric constant of the solvent. The derivation of Eq 10 from Eq 4 is described in the SI(S1 Text). A solution to a more complicated scenario can also be derived numerically[49].

In addition to the flux rate *k(r)* across spheres, the MFPT that a particle remains within a certain domain of space can also be obtained. For a system that obeys Smoluchowski theory, Eq 11 describes how the MFPT relates to a stationary distribution in that domain.

$$\tau =\frac{N}{J}=\frac{\int_{V}^{}udV}{D\Sigma_{i=1}^{k}\int_{A_{i}}^{}(\nabla u)dA_{i}}$$ (11)

Where 〈*τ*〉 is the MFPT, *N* is the total number of particles present in the system, *J* is the total flux of particles across all absorbing boundaries at any given time, *u* is the stationary distribution of particles, *V* is the volume of the system, *D* is the diffusion coefficient of the particle, *k* is the number of absorbing boundaries, and *i* is the index of a particular absorbing boundary *A*_*i*_[50].

## Materials and Methods

### Preparation of MD

All MD simulations were carried out using NAMD 2.9[51]. The MD FHPDs were made with the help of MDAnalysis[52]. All calculations were performed on the Gordon supercomputer at the San Diego Supercomputer Center, the Stampede supercomputer at the Texas Advanced Computing Center, and on local machines.

### Spherical receptor systems

MD simulations of the charged and uncharged spherical receptor simulations were prepared using a simple 40 Å x 40 Å x 40 Å TIP3P[53] water box, we placed a Cl^-^ in the center of the box for the charged spherical receptor. Both systems contain approximately 7600 atoms. Na^+^ and Cl^-^ parameters were obtained from the ions94 library of the AMBER ff03 forcefield[54]. The spherical receptor systems were minimized for 10000 steps to allow the water molecules to relax in relation to each other and to the Cl^-^. Both systems were then equilibrated for 20 ns at a constant temperature of 300K using the Langevin thermostat and constant pressure using the Langevin piston at 1 atm with a damping coefficient of 5 ps^−1^. The Cl^-^ was constrained to a stationary position in the center of the charged spherical receptor system.

**Fig 2:**
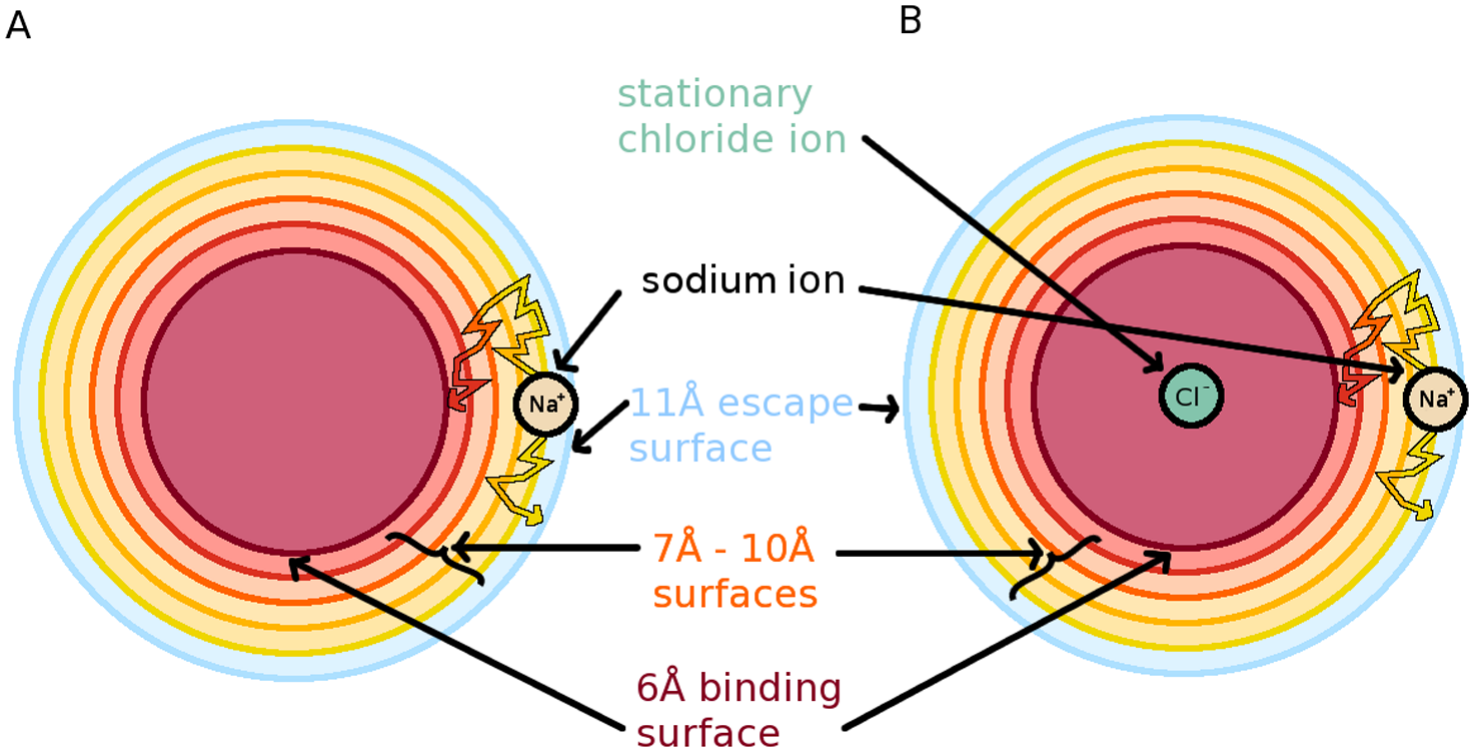
A cartoon depiction of the two spherical receptor systems drawn approximately to scale. The uncharged system in panel A has no central charged molecule. The charged system in panel B has a Cl^-^ constrained to the center of the spherical milestones. Both systems contain the escape milestone (light blue curves), four intermediate milestones (curves in shades of orange and yellow), and binding milestone (dark red curves). Two hypothetical paths are also depicted per system. The upper path shows a trajectory where Na^+^ diffuses within the simulation region, crossing surfaces and finally reacting with the 6Å spherical milestone. The bottom path shows Na^+^ diffusing across a few states before escaping to the 11Å milestone.

Following this equilibration, four copies were made of the systems, and a Na^+^ was placed at the milestones located at 7 Å, 8 Å, 9 Å and 10 Å from the center of the water box in the uncharged system (Fig 2), and from the Cl^-^ in the charged system. Two additional milestones were also placed at 6Å and 11 Å. Waters clashing with the Na^+^ were removed. The system was once again allowed to minimize for another 5000 steps to relax the waters around the ions. Then the system was heated in 10 K increments up to 350 K and then reduced back to 300 K at 2 ps intervals each at constant volume. Then, in order to obtain an ensemble distribution, the systems were simulated at constant temperature at 300 K at constant volume for 20 ns. To this point, all ions have been constrained. In order to obtain a FHPD, 900 position/velocity configurations were uniformly chosen between the 2 ns and 20 ns marks in the ensemble simulations. Velocities were reversed, and the trajectories were allowed to propagate backwards. If the trajectory struck another milestone before re-crossing the one it came from, that trajectory was considered part of the FHPD. All members of the FHPD were then allowed to proceed with their velocities in the forward direction. Each transition event was monitored for future milestoning analysis. Reverse simulations were carried out using a special plugin for NAMD 2.9[55], which allows velocities to be reversed at arbitrary timesteps.

For comparison with the milestoning results, brute-force MD simulations were run and Smoluchowski theory was used to estimate a *β*, k_on_, and MFPT for the spherical receptor systems. All brute-force MD simulations were set up with the same parameters as for milestoning above, except that the system was equilibrated for 40 ns and 10000 frames were sampled between the 20 and 40 ns time. Each of the 10000 simulations were started with the Na^+^ placed on the 10Å milestone and monitored for a crossing event at either the 6Å or the 11Å milestone. The value *β* was simply the number that crossed the 6Å milestone out of the total number of simulations. The MFPT was the average amount of time that all the simulations lasted before a crossing event.

### SOD system

MD force field (FF) parameters for SOD were obtained as a generous gift from Branco et. al.[56] The system was surrounded by a TIP3P[53] water box with 150 mM NaCl solution. The simulation contained approx. 44,000 atoms. The SOD system was then equilibrated for 80 ns at a constant temperature of 300 K using the Langevin thermostat and constant pressure using the Langevin piston at 1 atm using a damping coefficient of 5 ps^−1^.

**Fig 3:**
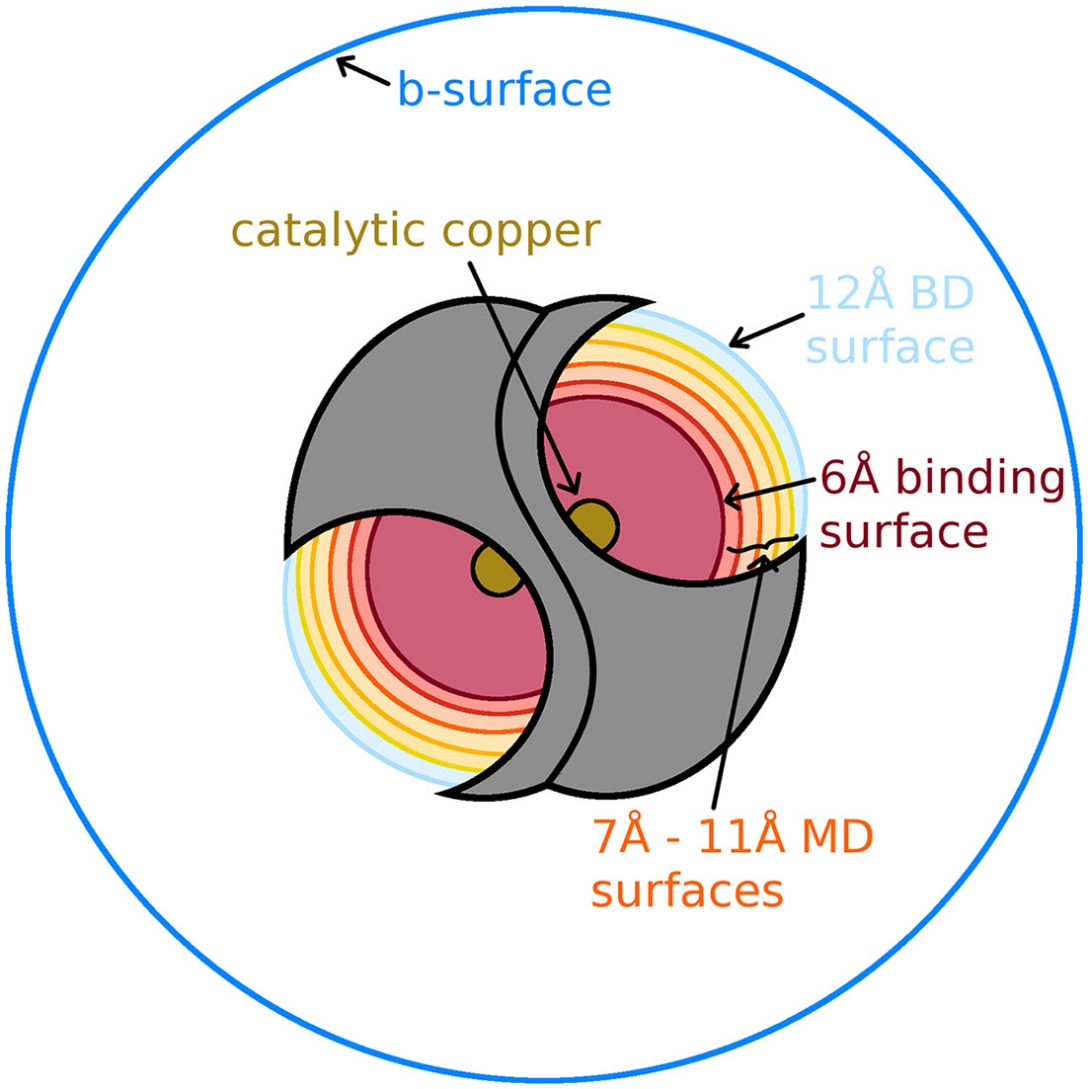
A cartoon depiction of the SOD system. The system has two binding sites, b-surface (dark blue circle), BD milestones (light blue curves), MD milestones (curves in shades of orange and yellow), and the binding milestone (dark red curves). The catalytic coppers at the center of the spherical surfaces are also depicted as tan circles in the bottom of each active site.

Following equilibration, ten copies were made of the apo system, and O_2_^−^ was inserted at eight different milestones (located at 4Å-11Å in 1Å increments) from each of the two copper ions in SOD’s two active sites, yielding a total of sixteen different milestones simulated (Fig 3). Waters clashing with O_2_^−^ were removed. The solvent molecules in the system were minimized for another 5000 steps to relax around the newly placed ions. Then the system was heated in 10 K increments up to 350 K and then reduced back to 295 K at 2 ps intervals each at constant volume. The protein and O_2_^−^ atom positions were constrained during the minimizations and heating/cooling. In order to obtain an ensemble distribution, the systems were simulated at a constant temperature of 300 K and constant volume for 200 ns each with an imposed harmonic “spring” force of 300 kcal mol^−1^ Å^−2^ that constrained O_2_^−^ close to a spherical milestone at each system’s proper distance from the SOD active site catalytic copper. In order to obtain a FHPD, 700 position/velocity configurations were uniformly chosen between the 60 ns and 200 ns marks in the ensemble simulations. Velocities were reversed, and the trajectories were allowed to propagate backwards in time. If the trajectory struck another milestone before recrossing the one it came from, that trajectory was considered part of the FHPD. The autoimage function in CPPTraj[57] was used to center the ligand in the waterbox before the reversal stage. All members of the FHPD were then allowed to proceed in the forward direction. Each crossing event was monitored for future analysis. The reversal phases were simulated using a custom plugin for NAMD 2.9[55].

### TnC system

FF parameters for TnC were prepared according to the protocol followed by Lindert et. al.[58] The system was surrounded by a TIP3P[53] waterbox with 100 mM KCl solution. The simulation contained approximately 27,000 atoms. The TnC system was then equilibrated for 100 ns at a constant temperature of 288 K using the Langevin thermostat and pressure using the Langevin piston at 1 atm using a damping coefficient of 5 ps^−1^.

**Fig 4:**
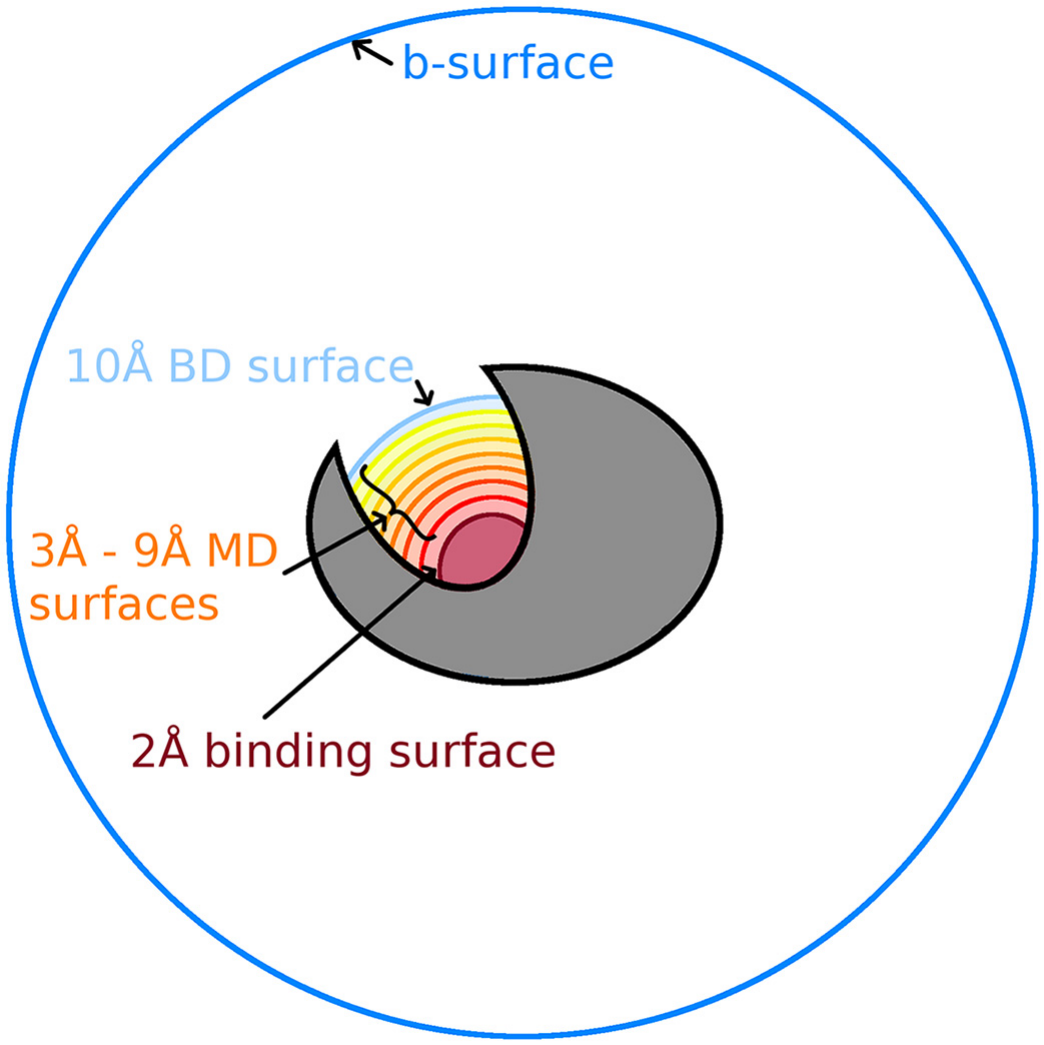
A cartoon depiction of TnC. The system contains a b-surface (dark blue circle), a BD surface (light blue curve), MD surfaces (curves in shades of orange and yellow), and a binding surface (dark red curve) located in the Ca^2+^ binding site (site II). Each curve represents a milestone.

Following this equilibration, twelve copies were made of the systems, and the Ca^2+^ was inserted on the binding side of the TnC site II loop at 1 Å increments from 2 Å to 9 Å from the center of mass of the alpha carbons of residues ASP 65, ASP 67, SER 69, THR 71, and GLU 76 (Fig 4). Waters clashing with Ca^2+^ were removed. The solvent molecules in the system were minimized for another 5000 steps to relax around the newly placed ions. Then the system was heated in 10 K increments up to 350 K and then reduced back to 295 K at 2 ps intervals each at constant volume. The protein and Ca^2+^ atoms were constrained during the minimizations and heating/cooling cycles. In order to obtain an ensemble distribution, the systems were simulated at a constant temperature of 300 K and constant volume for 100 ns each with an imposed harmonic force of 300 kcal mol^−1^ Å^−2^ that constrained Ca^2+^ close to the spherical surface at each system’s proper distance from the active site center of mass. In order to obtain a FHPD, 700 position/velocity configurations were uniformly chosen between the 30 ns and 100 ns marks in the ensemble simulations. The reversal phase of the TnC system was performed in an identical procedure as the SOD system.

### Preparation of BD

All Brownian dynamics simulations were performed using BrownDye[27] with desolvation forces and hydrodynamic interactions activated. All electrostatics calculations were performed using the Poisson-Boltzmann Equation solver APBS[59]. The solvent dielectric was left at the default of 78, and the permittivity of a vacuum was left at the default of 8.854×10^−12^ C^2^N^−1^m^−2^. All macromolecular dielectrics were set to 2, while the dielectrics of Ca^2+^ and O_2_^−^ were set to 1. A 6–12 hard sphere Lennard-Jones interaction was used. Simulations were distributed across 10 to 20 threads on a local computing node. The BrownDye program bd_top was used to prepare all systems for simulation. A phantom atom of zero charge and zero radius was placed at the center of the active sites in order to detect crossings of spherical milestones. The phantom atom has no effect on the dynamics, but is merely a convenient way to detect surface-crossing events. The BrownDye program nam_simulation was used for simulation, and the program compute_rate_constant was used to aid in the calculation of the association rate constants. Trajectories were processed using the BrownDye programs process_trajectories and xyz_trajectory in combination with in-house Python scripts.

### BD for SOD

A PQR file for SOD was prepared from the crystal structure PDB ID: 1CBJ[60] using LEaP[61] and DelEE[62] with the AMBER forcefield[63,64] and PROPKA^,47^ assigned protonation states at a pH of 7.0. A PQR file for O_2_^−^ was made by hand, with each oxygen given a partial charge of -0.5 and a radius of 1.5 Å. APBS[59] was then used to calculate the electrostatic field at 295 K and a NaCl concentration of 150 mM to approximate conditions used during the experimental measurement of k_on_ for SOD[65]. BrownDye was used to prepare and run 1×10^6^ BD simulations at 295 K with the ligand starting from a b-surface at ~61 Å from the SOD center of mass. Based on experimentally determined diffusion coefficient[66] of 1.5×10^−5^ cm^2^s^−1^, a hydrodynamic radius of 1.45 Å was used for O_2_^−^ in the simulations (See S1 Text). We used the Browndye default water viscosity of 1.00×10^−3^ kg m^−1^s^−1^ for all BD simulations of SOD. Reactions with both active sites, and also escape events were counted. 1000 configurations of ligand encounters with both active sites (12 Å from catalytic copper) were extracted to make two additional FHPD distributions. 1000 simulations were started from each configuration (2×10^6^ total). These were allowed to react with a surface further down the site (11 Å from the catalytic copper) react with the surface around the other site (12 Å from the other catalytic copper) or escape to infinity. All reaction and escape events were counted to construct the statistics of the transition kernel **K** and incubation time vector 〈*t*〉.

### BD for Troponin C

A PQR file for TnC was prepared from the NMR structure 1SPY[67]. Partial charges were assigned according the AMBER forcefield[61] using LEaP [63]and DelEE[62] and PROPKA[64] assigned protonation states at a pH of 7.0. A PQR file for Ca^2+^ was made by hand, given a charge of 2.0 e and an atomic radius of 1.14 Å. APBS[59] was then used to calculate the electrostatic field at 288 K and a KCl concentration of 100 mM to approximate conditions used during the experimental measurement of k_on_ and k_off_ for TnC[68]. A hydrodynamic radius of 3.0 Å was assigned based on an experimentally determined diffusion coefficient[69] of 6.73×10^−6^ cm^2^s^−1^ at 291 K (See SI S1 Text). BD simulations of TnC used an experimentally determined water viscosity of 1.138×10^−3^ kg m^−1^s^−1^ at 288 K[70]. BrownDye was used to prepare and run 1×10^6^ BD simulations at 288K with the ligand starting from a b-surface at ~57 Å from the TnC center of mass. Diffusion to the active site surface, and escapes were counted. 1000 configurations of ligand encounters with the active site (10 Å from binding site center of mass of residues ASP 65, ASP 67, SER 69, THR 71, and GLU 76) were extracted to make a FHPD distribution. 1000 simulations were started from each configuration (1×10^6^ total). These were allowed to react with a surface further down the site (7 Å from binding site center) or escape to an infinite distance. All reaction and escape events were counted to construct the milestoning model.

### Theoretical calculations

For our spherical receptor calculations, we used a dielectric of 92 to mimic the dielectric of TIP3P water[71], a permittivity of 8.854×10^12^ C^2^N^−1^m^−2^, and a diffusion coefficient[69] of 1.33×10^−5^ cm^2^s^−1^ for Na^+^. Although the dielectric of 92 for water is obtained from MD and was not experimentally measured, the spherical receptors were intended more for demonstration purposes rather than physical realism, and a dielectric of 92 was chosen in an attempt to allow the values obtained using Smoluchowski theory to match what we observe in the brute-force and milestoning MD simulations.

The rate constants *k(a)*, *k(b)*, and *k(q)* were calculated using Eq 9 for the uncharged spherical receptor and Eq 10 for the charged spherical receptor for the reaction surface, b-surface, and q-surface, respectively. The rate constant *k(a)* is the theoretical model of the spherical receptor association. For comparison, we deduced *k(a)* using only *k(b)*, and *k(q)* by using a transition matrix **K** obtained from monitoring transitions of the spherical receptor systems in a series of MD simulations. A binding probability *β* was calculated using Eq 8. The k_on_ for each spherical receptor system was calculated using Eq 12.

$$k_{on}=k\left(b\right)\left(\frac{\beta }{1-\left(1-\beta \right)\left(\frac{k\left(b\right)}{k\left(q\right)}\right)}\right)$$ (12)

The MFPT represents the mean time taken by a particle started on the b-surface and allowed to diffuse before touching either the reaction surface or the q-surface. The MFPT was calculated using Eq 12. The values *k(b)* and *k(q)* are obtained using Eq 9 or Eq 10, depending respectively on the absence of presence of a receptor charge.

### Milestoning calculations

For each system, the milestoning calculations were performed using custom scripts that used Numpy 1.7, Scipy 0.9.0 and the GNU Parallel tool[72].

## Results

Using Smoluchowski theory, milestoning, and brute force MD simulations, the probability *β* of each system starting on the b-surface and continuing on to touch the reaction surface is listed in Table 1 along with the resulting k_on_. The MFPT is also listed for the spherical receptor systems. It is important to note that Smoluchowski theory, as we implemented it, makes use of an idealized model of the system where waters are not modeled explicitly, and therefore the MD and milestoning implementations have different diffusion properties from the theoretical model.

**Table 1:** Computationally and theoretically determined results for the charged and uncharged spherical receptor system.

Spherical Receptor System	Method	*β*	k_on_ (M^−1^s^−1^)	MFPT (ps)
Uncharged	Milestoning MD	0.113±0.012	5.9±0.9×10^9^	7.4±0.5
	Theoretical model (using Eq 7)	0.12	6.039×10^9^	13.5
	Brute-force MD	0.114±0.013	5.9±0.9×10^9^	7.2±0.3
Charged	Milestoning MD	0.127±0.013	9.1±1.3×10^9^	7.6±0.4
	Theoretical model (using Eq 9)	0.146	9.589×10^9^	14.2
	Brute-force MD	0.135±0.012	9.3±1.2×10^9^	8.3±0.3

All simulations were carried out in a dilute aqueous environment. *β* is the probability of a particle starting on the b-surface to reach the bound state before touching the q-surface. MFPT refers to the mean first passage time of a particle started on the b-surface to reach either the reaction surface or the q-surface.

**Fig 5:**
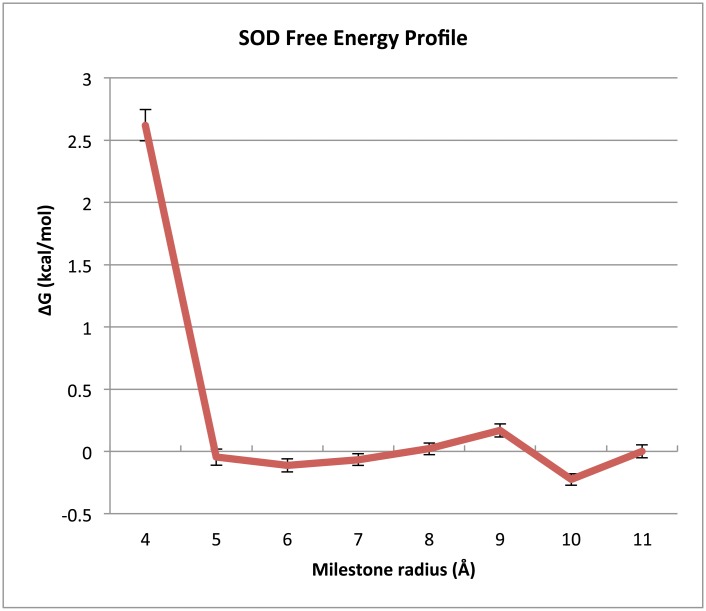
SOD system free energy profile. This plot depicts the free energy in kcal/mol at each milestone along the reaction coordinate in the SOD system relative to the 10Å milestone, the nearest to the bulk solution. These free energies were computed using milestoning theory according to Eq 12. A slight local minimum occurs at 6Å and we assume this to be the bound state.

Using the stationary probabilities obtained with milestoning of SOD, Eq 5, Eq 6 and Eq 13 below, we constructed a free energy profile for the approach of O_2_^−^ to the SOD binding site (Fig 5) setting the 10Å milestone to zero energy as a reference.

$$\Delta G_{i}=-k_{b}Tln\left(\frac{p_{i,stat}}{p_{ref,\;\;stat}}\right)$$ (13)

Where *ΔG*_*i*_ is the estimated free energy of milestone *i*, *k*_*B*_ is Boltzmann’s constant, *T* is temperature, and *p*_*i*,*stat*_ and *p*_*ref*,*stat*_ are the stationary probabilities of milestone *i* and the reference milestone at 10Å, respectively, obtained using Eq 6.

Luty et. al. assumed that the bound state was a spherical surface of radius 6Å centered on the catalytic copper. This location does appear to have a shallow local minimum at 6Å in the free energy as depicted in Fig 5. Because Luty et. al. assumed that the 6Å sphere was the bound state, and because it is the location of a shallow local minimum in the free energy profile in Fig 5, we assume that the catalytic copper and O_2_^−^ are in a close enough proximity to one another at 6Å that the rapid and essentially irreversible dismutation reaction occurs. Table 2 lists the estimated k_on_ rate constants obtained in this study for the SOD system.

**Table 2:** Computationally and experimentally determined k_on_s for SOD by us and others.

Researchers	k_on_ (M^−1^s^−1^)	Temp. (K)	Ion Conc. (mM)	Method
This study	1.23±0.09×10^9^	295	150 NaCl	MD/BD/milestoning
Cudd, et. al.	8.5×10^8^	300	140 NaCl	Pulse-Radiolysis
Argese, et. al.	1.6×10^9^	295	160 NaClO_4_	Polarographic method of catalytic currents & NMR
Luty, et. al.	1.62±0.86×10^9^	300	0	MD/BD, 7-state MSM

The experimental value that this study attempted to emulate[65] measured a k_on_ is listed along with an additional experiment[73] and the k_on_ that Luty et. al. [30] determined for SOD using different simulation conditions and model setup.

**Fig 6:**
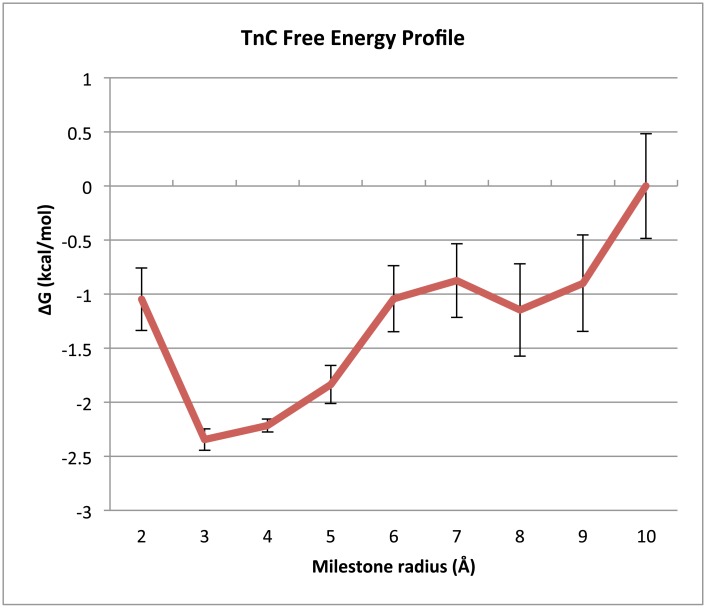
TnC system free energy profile. This plot depicts the free energy profile of TnC binding (in kcal/mol) relative to the 10Å milestone at each milestone along the reaction coordinate of the TnC system. These free energies were computed using milestoning theory according to Eq 12. The lowest relative free energy is at the 3Å milestone, the location we assume to be the bound state of the TnC system.

As with the SOD system, we used the stationary probabilities obtained with milestoning of TnC, Eq 5, Eq 6 and Eq 12 to construct a free energy profile for Ca^2+^ in its approach to the TnC binding site (Fig 6) with the 10Å milestone free energy as the reference. According to this profile, the lowest energy state is located at 3Å from the binding site center. We assume that when the Ca^2+^ has reached this distance, it is in the bound state. We use a 3Å binding surface for all subsequence milestoning calculations on TnC. The estimated k_on_ rate constants for the TnC system are listed in Table 3.

**Table 3:** Computationally and experimentally determined k_on_s for TnC by us and others.

Researchers	K_on_ (M^−1^s^−1^)	Temp. (K)	Ion Conc. (mM)	Method
This study	9.0±2.0×10^8^	288	100 KCl	MD/BD/milestoning
Tikunova, et. al.	1.7±0.3×10^8^	288	90 KCl	Stopped-flow
Hazard, et. al.	2–4×10^8^	277	90 KCl	Stopped-flow
Ogawa, et. al.	>4.0×10^7^	293	100 KCl	Stopped-flow

The k_on_ we predicted is listed along with that of the experimental value that this study attempted to emulate[68], along with additional experimental k_on_s[74,75].

**Fig 7:**
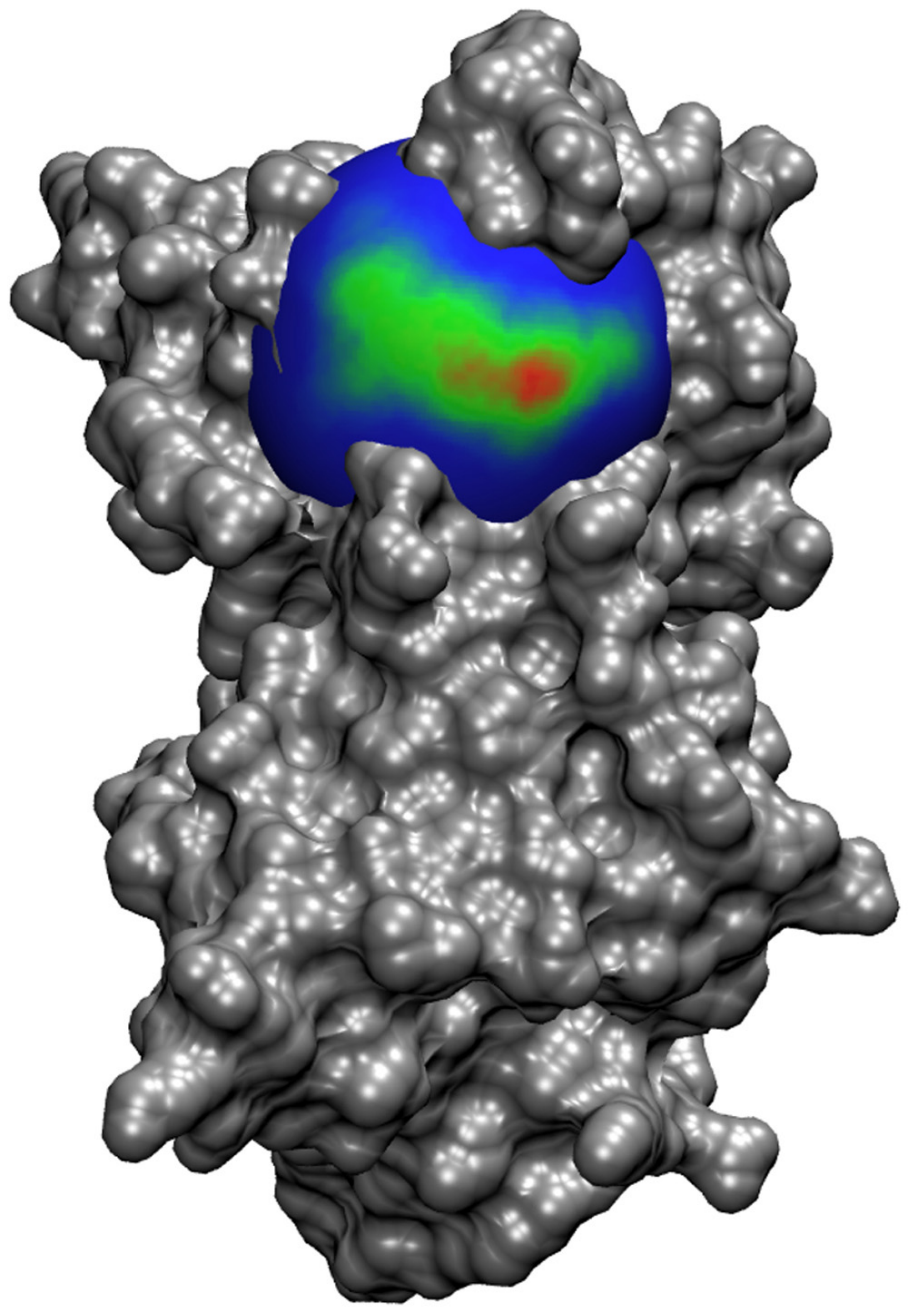
The FHPD for O_2_^−^ encounter on the 12Å around the active site of SOD. Blue indicates zero crossing events per square Å, and the color scale increases to red, indicating up to 1.2×10^5^ crossing events among all 1×10^6^ simulations. The distribution suggests that O_2_^−^ approaches directly from the solvent instead of approaching laterally from another portion of the protein surface. The image was generated using VMD[76] with an MSMS surface[77].

**Fig 8:**
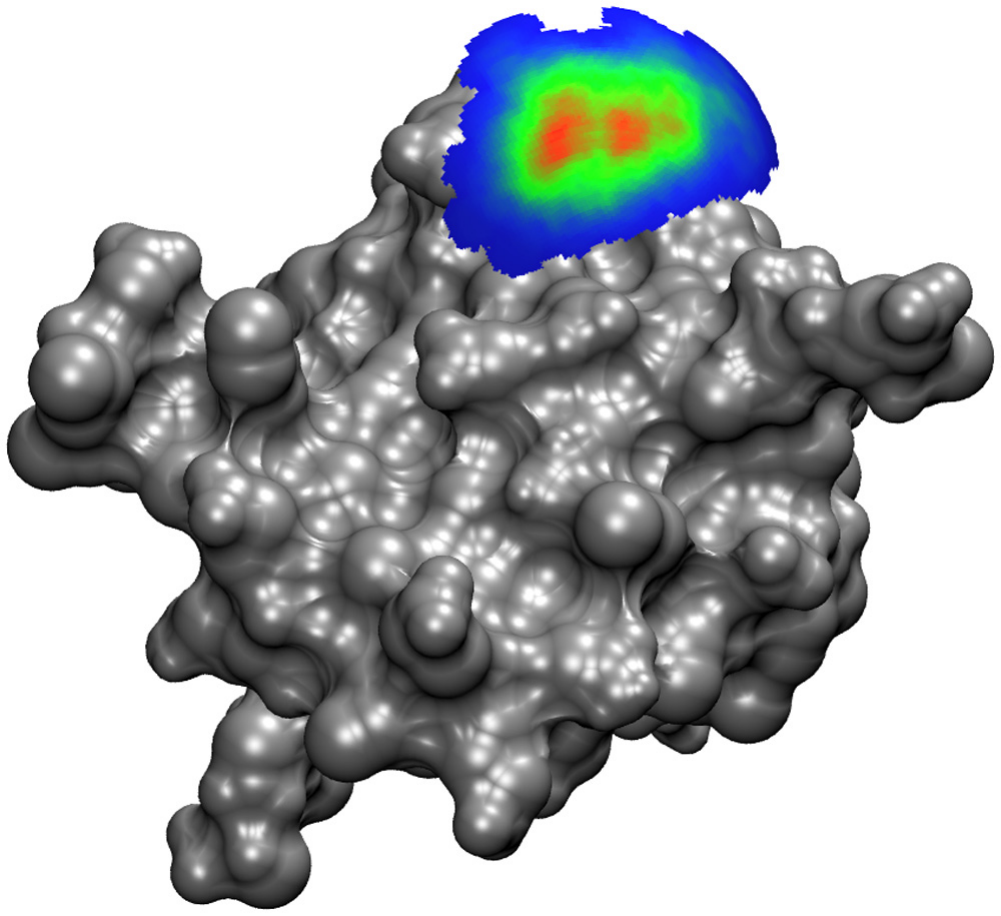
The FHPD for Ca^2+^ encounter on the 10Å around the binding site of TnC. Blue indicates zero crossing events per square Å, and the color scale increases to red, indicating up to 8.8×10^4^ crossing events among all 1×10^6^ simulations. The distribution suggests that Ca^2+^ approaches the site directly from the solvent instead of approaching laterally from another portion of the protein surface. The region of the sphere where no ligands crossed was removed to reveal the site II loop over the binding site, though the binding site itself is concealed by the FHPD. The image was generated using VMD[76] with an MSMS surface[77].

In addition to the calculation of k_on_ rate constants, the milestoning models and distributions across the states can be used to visualize the path of the ligand in its approach to association within the binding site. The FHPD for SOD at 12 Å is displayed in Fig 7 and the FHPD for TnC at 10 Å is displayed in Fig 8.

### Computational performance

The total computational cost of all systems simulated in this study for both MD and BD was approximately 65,000 CPU hours. Computational costs of each simulated system and simulation regime are listed in Table 4. The cost of performing all non-simulation calculations was negligible. Table 4 includes all computer time spent on the supercomputer as well as on local machines. The *β*, k_on_s and error estimates for all systems were well converged and are reported in the SI (S2–S9 Figs).

**Table 4:** The computational cost of calculating kinetics for each system using milestoning.

System	Cost of MD (CPU- hours)	Length of MD (ns)	Cost of BD (CPU- hours)	Computer used for MD	Computer used for BD	Cost of Brute-force MD (CPU- hours)
Uncharged spherical receptor system	~600	~100	-	Linux desktop	-	~1350
Charged spherical receptor system	~600	~100	-	Linux desktop	-	~1450
SOD	~53,000	~1630	~100	Stampede Supercomputer	Linux desktop	-
TnC	~5100	~900	~100	Gordon Supercomputer	Linux desktop	-

BD simulations were not run for the spherical receptor systems, so no costs are listed. Also, brute force MD simulations were not run for SOD and TnC.

## Discussion

### Idealized systems

The k_on_ calculated using milestoning for the uncharged spherical receptor system matches within 3% to the theoretically determined value and 0.3% to the brute-force MD value. These estimates are well within the bounds of uncertainty introduced by the milestoning model. As a system that can diffuse freely without forces or solvation shells, it is expected that Smoluchowski theory would yield such a close result to simulation. This similarity to a value obtained using well-established theory is a good validation of our basic methodology. The large difference between the MFPT predicted by theory and the MFPTs predicted by milestoning and brute force MD could be due to a difference between the experimentally measured diffusion coefficient of Na^+^, and the diffusion coefficient that is observed in an MD simulation using the AMBER forcefield.

The k_on_ calculated using milestoning for the charged spherical receptor system differs by 13% from the k_on_ predicted by Smoluchowski theory and by only 6% from the k_on_ obtained by brute force MD simulation. This difference between the simulation-obtained values and the value obtained by theory is likely due to effects caused by the explicit solvent in our simulations, for which this simple implementation of Smoluchowski theory does not account. Very likely, solvation shells have formed around the Cl^-^ placed in the center of the system, as well as the diffusing Na^+^. Solvation shells create unevenness in the potential of mean force and the position-dependent diffusion coefficient of Eq 4. As such, using Coulomb’s law for the electrostatic potential and a constant diffusion coefficient may not be sufficiently valid assumptions for ions in solution at such close proximity. Previous studies on close NaCl ion pair interactions in dilute solvent show oscillations in the mean force potential of the interionic distance that extend several molecular layers into the solvent[78–80]. Accounting for these factors and using an alternative solution to Eq 4 would likely result in a calculated value much closer to what we obtained using milestoning and the brute force MD. The fact that the milestoning results and the brute-force MD results are so similar supports the validity of the milestoning methodology. Similarly, with the charged receptor, the large difference in the MFPT predicted by theory and the MFPTs predicted by milestoning and brute force MD could be due to a difference between the experimentally measured diffusion coefficient of Na^+^, and the diffusion coefficient that would be observed in an MD simulation using the AMBER forcefield. It could also be due to the same effects observed on *β* caused by the aforementioned solvation shells.

### Superoxide Dismutase (SOD)

SOD is an enzyme found in a wide variety of organisms[73]. It is a homodimer that makes use of a catalytic copper bound in its active site to catalyze the dismutation of the superoxide ion O_2_^−^ into O_2_ and H_2_O_2_ [65,73]. SOD was the subject of many early enzymology experiments[81] and ligand-receptor binding simulations[38,82].

The SOD k_on_ estimated using milestoning is within a factor of ~1.5 of the experimentally measured k_on_ that this study attempted to emulate. Although this value falls outside the uncertainty bracket calculated for the milestoning model, it is still within the range of k_on_s measured in other studies[73]. The k_on_ we calculated is also close to the value obtained by Luty, et. al. in their seminal study of SOD kinetics[30]. It is well understood that a higher salt concentration slows the rate of O_2_^−^ binding to SOD[73]. Therefore, the k_on_ measured in this study is likely smaller than the value measured by Luty, et. al. because they simulated MD and BD with a solvent salt concentration of zero. The discrepancy could also be due to differences used by Luty et. al. in their implementations of atomic constraints on the protein, different boundary conditions in the MD phase, and the lack of desolvation forces in the BD phase.

While it is not clear how much error is introduced by using an equilibrium distribution across the milestones, our use of a FHPD should, theoretically, provide a more accurate treatment due to its consistency with formal milestoning theory[18,19]. The insertion of additional states in the MD region also allowed us to obtain much better sampling of transition events than would be available for a comparable computation time if the MD region had been composed of only a single milestone. The FHPD of SOD at 12Å (Fig 7) indicates that O_2_^−^ approaches directly from the solvent and does not seem to sample much of the protein surface before entering the active site. Although a k_on_ has already been obtained for this system by Luty et. al. using similar methods, our approach offers a number of key improvements and more closely resembles the experimentally obtained rate constant; both insofar as the conditions that the system was exposed to, as well as the final result.

### Troponin C (TnC)

In order to try this milestoning method on a new system, we also calculated the k_on_ of TnC. The troponin complex is a set of proteins that regulates muscle contraction in skeletal and cardiac muscles[67,68,75]. One of the subunits, TnC is attached to the thin filaments of a muscle fiber, and regulates the binding of Ca^2+^ to the N-terminal domain of TnC[83]. Ca^2+^ binding triggers changes within the complex, allowing myosin to latch onto the thin filaments and induce muscle contraction. TnC has been extensively studied due to its critical involvement with heart function and failure, and has been marked as a therapeutic target in heart disease and other disorders[68].

Our method is able to determine the k_on_ to a value that is within a factor of ~5 of the experimentally measured k_on_ that our study attempted to emulate. Although this discrepancy falls outside of both the experimental uncertainty as well as the uncertainty of the milestoning calculation, the value is not unreasonable when compared to k_on_ values measured in other studies[74,75]. The FHPD of TnC at 10 Å (Fig 8) indicates that Ca^2+^ approaches directly from the solvent, probably due to the high desolvation penalty incurred when the highly charged Ca^2+^ is removed from its aqueous environment. The surface map seems to indicate two close but distinct minima on the FHPD, suggesting that Ca^2+^ may have two possible routes to binding (Fig 8).

In total, the entire project, including all simulations of all systems analyzed in this study, cost approximately 65,000 hours of CPU usage. The vast majority of this computation was spread across hundreds or thousands of cores at any one time due to the highly parallel nature of milestoning. The total length of MD simulation for our systems required anywhere between 100 and 1600 ns of total MD time each with relatively low uncertainty due to the high rate of sampling along the milestones leading to binding. The cost is significantly less per target than brute force MD simulations run in past studies to observe kinetic events while yielding similar or superior resemblance to experiment[4,5], which were indicated to require between 600 and 15000 ns of MD simulation to achieve even just a single binding event, with some simulations never even yielding a binding event.

Our multiscale MD-BD-milestoning method offers many advantages; yielding predictive k_on_ estimates for biologically relevant molecular systems within a range of experimental measurements at a cost much less than brute-force MD alone and at accuracy much greater than could be obtained using BD alone. This method also benefits from high parallelism due to the spread of MD computation across multiple states. Given a large number of cores and sufficient CPU hours, the MD portion of the calculation can be completed rapidly. Another advantage of this method is its flexibility, giving the user the ability to adjust the cost-to-accuracy balance by performing additional simulation and adding trajectory samples to increase result convergence. Theoretically, this milestoning framework could be used to investigate any biomolecular association where MD and BD simulation methods can adequately model the process. Estimating the binding kinetics between proteins, DNA, small molecules, or any combination thereof ought to be possible, assuming that sufficient sampling effectively constructs the proper FHPD.

**Fig 9:**
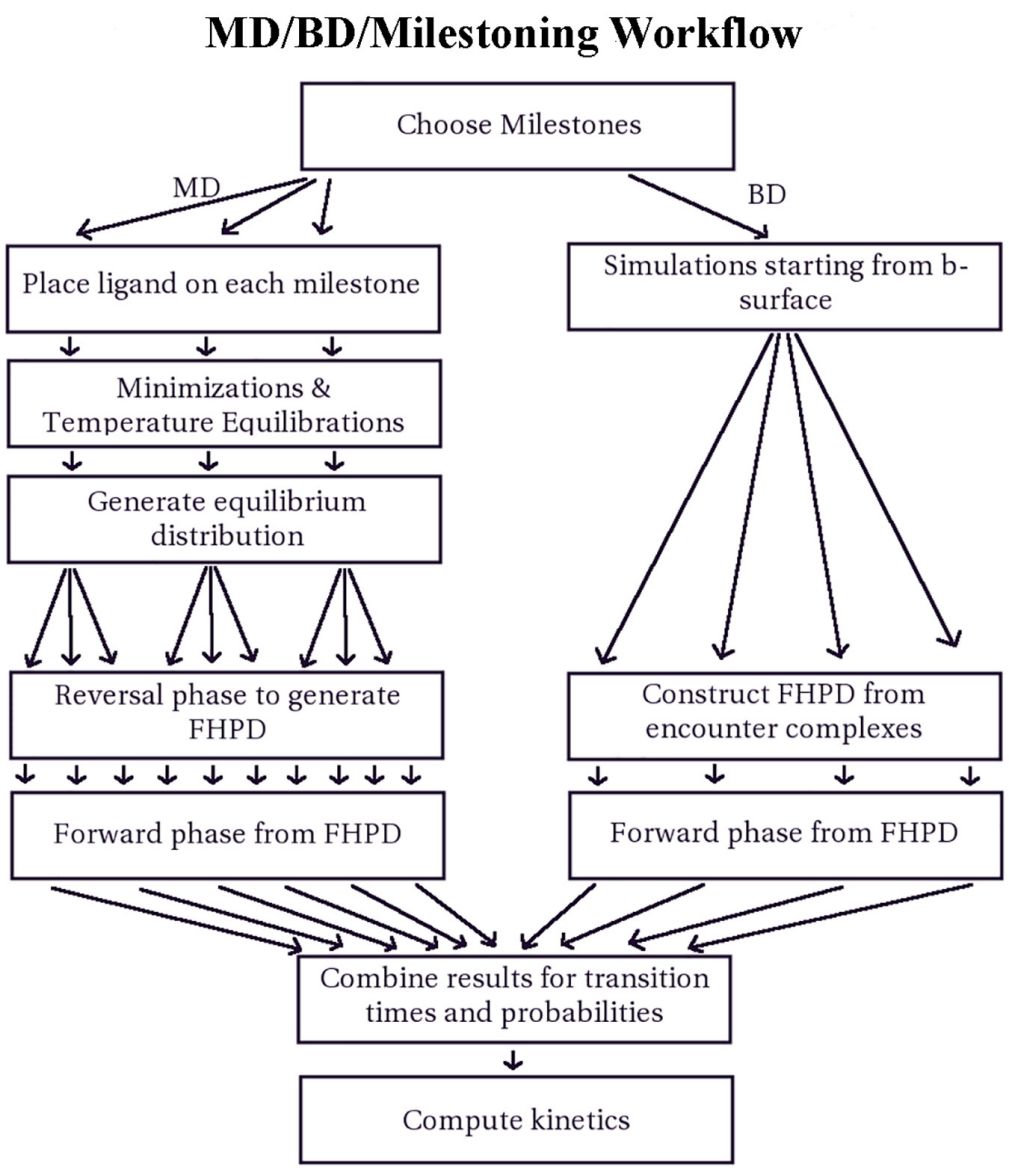
A schematic of the MD/BD/milestoning workflow. This workflow illustrates how MD and BD simulations are prepared, run, and unified using milestoning. The number of arrows in this workflow are intended to be metaphorical, and do not correspond to actual numbers of simulation instances. Splits into multiple arrows indicate that a large number of simulations are commenced from a distribution generated at the previous step.

The main disadvantage of this method lies in its complexity of concept and implementation (Fig 9), particularly in the maintenance of large numbers of simultaneous simulations. However, with sufficiently robust software-based automation, the burden of maintaining many parallel instances of simulation, as well as simulation preparation and analysis, can be greatly reduced. Another disadvantage of the milestoning framework is that the simulations are still relatively expensive at this time; requiring a supercomputer or cluster to obtain sufficient sampling within a reasonable time frame, although GPU-based MD could potentially alleviate this burden.

### Conclusions

We present a new method to estimate kinetic rates. This method uses milestoning to leverage the strengths and minimize the weaknesses of MD and BD, thereby offering an efficient, high-accuracy estimation of k_on_ rate constants. This multiscale method has been successfully used to estimate the k_on_ rate constant for both idealized and realistically sized, biologically relevant systems. Our work demonstrates that milestoning can be used to obtain kinetic quantities of interest with a high resemblance to experiment. We anticipate that this multiscale approach can be used to determine rate constants of interest as well as system-specific binding details that are applicable to drug discovery, biomolecular modeling, and protein-ligand interactions.

## Supporting Information

S1 Text Supporting Information. (DOCX)

S1 Fig Plot illustrating the sampling of rate matrix. (TIF)

S2 Fig Convergence of error estimate for the β of the uncharged spherical receptor. (EPS)

S3 Fig Convergence of error estimate for the β of the charged spherical receptor. (EPS)

S4 Fig Convergence of error estimate for the β of SOD. (EPS)

S5 Fig Convergence of error estimate for the β of TnC. (EPS)

S6 Fig Convergence of the results of the uncharged spherical receptor system. (EPS)

S7 Fig Convergence of the results of the charged spherical receptor system. (EPS)

S8 Fig Convergence of the results of SOD system. (EPS)

S9 Fig Convergence of the results of TnC system. (EPS)
