# Supplementary material for: Multiscale Estimation of Binding Kinetics Using Brownian Dynamics, Molecular Dynamics and Milestoning
Source: PLoS Comput Biol. 2015 Oct 27;11(10):e1004381. doi: 10.1371/journal.pcbi.1004381 (PMC4624728; doi:10.1371/journal.pcbi.1004381)
Supplement: S1 Text — (DOCX) [file pcbi.1004381.s001.docx]

**Supporting Information**

**Hydrodynamic Radius**

The hydrodynamic radius *a* of a molecule relates to its diffusion coefficient *D* according to S1 Eq.[[1](#_ENREF_1)]

|  | $D=\frac{k_{B}T}{6\pi\eta a}$ | (S1) |
| --- | --- | --- |

Where *T* is the temperature, *k_B_* is Boltzmann’s constant, and $\eta$ is the viscosity of the solvent.

**Calculation of error for Milestoning**

The statistical error of all milestoning calculations can be estimated by generating a distribution of rate matrices according to S2 Eq.{Majek, 2010 #191;Vanden-Eijnden, 2008 #192}.

|  | $p\left( \mathbf{Q} \vert\left\{ N_{\alpha\gamma}, \left\langle t \right\rangle_{\alpha} \right\} \right)\propto\prod_{\alpha} \prod_{\gamma\neq\alpha} q_{\alpha\gamma}^{N_{\alpha\gamma}}e^{-q_{\alpha\gamma}N_{\alpha}\left\langle t \right\rangle_{\alpha}}P(\mathbf{Q})$ | (S2) |
| --- | --- | --- |

Where **N** is a count matrix whose element *N_αγ_* is equal to the number of times in the milestoning simulation that the system started at milestone α and ended at milestone *γ*, $\left\langle\mathbf{t} \right\rangle$ is the incubation time vector whose element $\left\langle t \right\rangle_{\alpha}$ is the average amount of time a system started at milestone α spends before crossing another milestone. *P(***Q***)* is a prior probability distribution that, in this case and typically, we set to uniform density. **Q** is a rate matrix whose nondiagonal elements q_αβ_ can be used to reconstruct the transition kernel **K** and the incubation time vector $\left\langle\mathbf{t} \right\rangle$ found in Eqs. 5-7 according to S3a and S3b Eqs.

|  | $K_{\alpha\beta}=\frac{q_{\alpha\beta}}{\sum_{\gamma\neq\alpha} q_{\alpha\gamma}}$  $\left\langle t \right\rangle_{\alpha}=\frac{1}{\sum_{\gamma\neq\alpha} q_{\alpha\gamma}}$ | (S3a)  (S3b) |
| --- | --- | --- |

The diagonal elements of **Q** are defined as: $q_{\alpha\alpha}=-\sum_{\alpha\neq\beta} q_{\alpha\beta}$. All non-diagonal elements *q_αβ_* > 0 and all diagonal elements *q_αα_* < 0.

By extracting a large number (hundreds or thousands) of matrices from this distribution, and performing the necessary milestoning calculations with each of them, a distribution of any of the results can be found, giving an estimate of the error for each result by finding a standard deviation of the distribution.

We used a nonreversible element shift Monte Carlo algorithm to sample the posterior probability in S2 Eq. Inspired by an algorithm used to compute the error of Markov state models (MSM){Noe, 2008 #321}, our algorithm is defined below:

**Algorithm for sampling rate matrices.** To sample the distribution S2 Eq., a metropolis criterion is defined that evaluates whether to take a proposed step in **Q** space. The first rate matrix **Q***** is the matrix that maximizes the likelihood of S2 Eq.

|  | $q_{\alpha\beta}^{*}=N_{\alpha\beta}/(N_{\alpha}t_{\alpha})$ | (S4) |
| --- | --- | --- |

Given a proposed matrix **Q***’* and a current matrix **Q**, the probability of accepting that member of the distribution is defined as:

|  | $p_{accept}=\frac{p(\mathbf{Q}^{\mathbf{'}}\vert\left\{ \mathbf{N},\left\langle\mathbf{t} \right\rangle\right\})}{p(\mathbf{Q}\vert\left\{ \mathbf{N},\left\langle\mathbf{t} \right\rangle\right\})}$ | (S5) |
| --- | --- | --- |

A proposed change Δ relates the difference between an element of **Q** and **Q***’*.

|  | $q_{\alpha\beta}^{'}=q_{\alpha\beta}+ \Delta$  $q_{\alpha\alpha}^{'}=q_{\alpha\alpha}- \Delta$ | (S6a)  (S6b) |
| --- | --- | --- |

The proposed change must ensure that all non-diagonal elements of **Q***’* remain positive, and that all diagonal elements remain negative. Thus, Δ is drawn from an exponential distribution on the range:

|  | $\Delta\in\left[ -Q_{\alpha\beta} \right.,\left. \infty\right)$ | (S7) |
| --- | --- | --- |

With a mean value at zero. Finally,

|  | $p_{accept}=\frac{p(\mathbf{Q}^{'}\vert\left\{ \mathbf{N},\left\langle\mathbf{t} \right\rangle\right\})}{p(\mathbf{Q}\vert\left\{ \mathbf{N},\left\langle\mathbf{t} \right\rangle\right\})}=\left( \frac{q_{\alpha\gamma}+\Delta}{q_{\alpha\gamma}} \right)^{N_{\alpha\beta}}\frac{e^{-{(q}_{\alpha\gamma}+\Delta)N_{\alpha}\left\langle t \right\rangle_{\alpha}}}{e^{-q_{\alpha\gamma}N_{\alpha}\left\langle t \right\rangle_{\alpha}}}$ | (S8) |
| --- | --- | --- |

**Example 1**. To support that the above workflow is correct, we have constructed a simple system to validate it. S1 Fig. compares the distributions of the off-diagonal elements of a 2x2 rate matrix computed using the count matrix and incubation time vector below:

|  | $\mathbf{N}=\left( \begin{matrix} 0 & 12 \\ 30 & 0 \end{matrix} \right) \left\langle\mathbf{t} \right\rangle=\left( \begin{matrix} 500 \\ 150 \end{matrix} \right)$ |  |
| --- | --- | --- |

A set of 1×10^6^ rate matrices were generated stochastically using the algorithm outlined above and the off-diagonal elements were binned to generate the 2-dimensional histogram in panel (a) of S1 Fig. For comparison, an analytic probability distribution was constructed in panel (b) of S1 Fig. by simply plotting the likelihood L for sampling that point in **Q**-space given the count matrix **N** and incubation vector $\left\langle\mathbf{t} \right\rangle$.

|  | $L(q_{01},q_{10})= q_{01}^{N_{01}}q_{10}^{N_{10}}e^{-q_{01}N_{0}\left\langle t \right\rangle_{0}-q_{10}N_{1}\left\langle t \right\rangle_{1}}$ |
| --- | --- |

The high degree of similarity between the two plots of S1 Fig. supports the correctness of the algorithm. These computations were done using a custom script.

**S1 Fig. Plot illustrating the sampling of rate matrix**. The rate matrix was constructed using count matrix $\mathbf{N}=\left( \begin{matrix} 0 & 12 \\ 30 & 0 \end{matrix} \right)$ and the incubation time vector $\left\langle\mathbf{t} \right\rangle=\left( \begin{matrix} 500 \\ 150 \end{matrix} \right)$. 1×10^6^ **Q** matrices were sampled from S2 Eq. using these criteria and the off-diagonal elements were binned into a histogram to generate the plot in panel (a). The plot in panel (b) was generated analytically for comparison.

**Error Estimate Convergence**

For the spherical receptor systems, 1×10^7^ matrices were generated from the distribution in S2 Eq. Of these, every 1000 were skipped, and the remaining 1000 were used to construct the error estimates for the spherical receptor systems. S2 Fig. and S3 Fig. were also constructed from those same samples to demonstrate convergence. For both SOD and TnC, only 100 matrices were skipped between samples, though 1000 total were sampled to construct S4 Fig. and S5 Fig.

**S2 Fig. Convergence of error estimate for the β of the uncharged spherical receptor.** The estimate is well converged before the full 1000 matrices have been sampled.

**S3 Fig. Convergence of error estimate for the β of the charged spherical receptor.** The estimate is well converged before the full 1000 matrices have been sampled.

**S4 Fig. Convergence of error estimate for the β of SOD**. The estimate is well converged before the full 1000 matrices have been sampled.

**S5 Fig. Convergence of error estimate for the β of TnC**. The estimate is well converged before the full 1000 matrices have been sampled.

**Results Convergence:**

The convergence of β, the mean first passage time (MFPT), and the k_on_ for each system was calculated by progressively increasing the number of MD trajectories from each milestone included in the milestoning computation (S6-S9 Figs.).

**S6 Fig. Convergence of the results of the uncharged spherical receptor system.** The β convergence is displayed in blue, the MFPT (×10^11^ s) in red, and the k_on_ (×10^-10^ M^-1^s^-1^) in green.

**S7 Fig. Convergence of the results of the charged spherical receptor system.** The β convergence is displayed in blue, the MFPT (×10^11^ s) in red, and the k_on_ (×10^-10^ M^-1^s^-1^) in green.

**S8 Fig. Convergence of the results of SOD system.** The convergence of β is displayed in red, the k_on_ (×10^-10^ M^-1^s^-1^) in blue.

**S9 Fig. Convergence of the results of TnC system.** The convergence of β is displayed in red, the k_on_ (×10^-10^ M^-1^s^-1^) in blue.

**Derivation of Eq. 9**

Eq. 4 describes the solution to the diffusion-convection equation for a charged particle diffusing around an absorbing spherical surface surrounded by a centrosymmetric force. We assume *D(r)* is a constant *D* and *U(r)* is defined by Coulomb’s law,

|  | $U\left( r \right)=\frac{Q_{c}Q_{s}}{4\pi\varepsilon_{0}\varepsilon_{r}r}$ | (S9) |
| --- | --- | --- |

where *Q_s_* is the charge of the diffusing particle, *Q_c_* is the charge in the center of the receptor sphere, *ε_0_* is the permittivity of a vacuum, *ε_r_* is the dielectric constant of the solvent, and *r* is the radius from the sphere center. Note that

|  | $\int_{b}^{\infty} r^{-2}e^{Cr^{-1}}dr=-\frac{1}{C}\left[ 1-e^{\frac{C}{b}} \right]$ | (S10) |
| --- | --- | --- |

where *C* is some constant. By assuming that

|  | $C=\frac{Q_{c}Q_{s}}{4\pi\varepsilon_{0}\varepsilon_{r}k_{B}T}$ | (S11) |
| --- | --- | --- |

we obtain Eq. 9.

**SI References:**

1. Mccrackin FL, Guttman CM, Akcasu AZ (1984) Monte-Carlo Calculations of the Hydrodynamic Radii of Polymers in Theta and Good Solvents. Macromolecules 17: 604-610.

2. Majek P, Elber R (2010) Milestoning without a Reaction Coordinate. Journal of Chemical Theory and Computation 6: 1805-1817.

3. Vanden-Eijnden E, Venturoli M, Ciccotti G, Elber R (2008) On the assumptions underlying milestoning. Journal of Chemical Physics 129.

4. Noe F (2008) Probability distributions of molecular observables computed from Markov models. Journal of Chemical Physics 128.
