# Supplementary figures and images for: Multiscale Estimation of Binding Kinetics Using Brownian Dynamics, Molecular Dynamics and Milestoning
Source: PLoS Comput Biol. 2015 Oct 27;11(10):e1004381. doi: 10.1371/journal.pcbi.1004381 (PMC4624728; doi:10.1371/journal.pcbi.1004381)

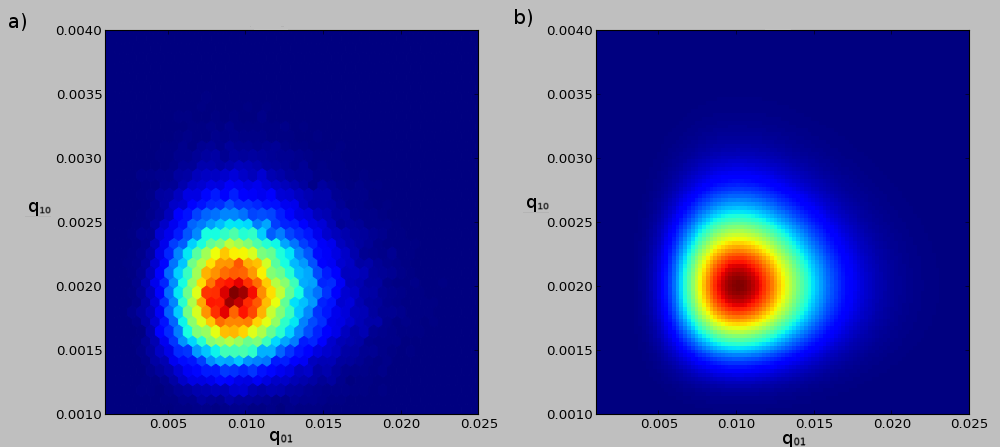

Supplement: S1 Fig — (TIF) [file pcbi.1004381.s002.tif]
